# A Versatile Class of Cell Surface Directional Motors Gives Rise to Gliding Motility and Sporulation in *Myxococcus xanthus*

**DOI:** 10.1371/journal.pbio.1001728

**Published:** 2013-12-10

**Authors:** Morgane Wartel, Adrien Ducret, Shashi Thutupalli, Fabian Czerwinski, Anne-Valérie Le Gall, Emilia M. F. Mauriello, Ptissam Bergam, Yves V. Brun, Joshua Shaevitz, Tâm Mignot

**Affiliations:** 1 Laboratoire de Chimie Bactérienne, CNRS UMR 7283, Aix-Marseille Université, Institut de Microbiologie de la Méditerranée, Marseille, France; 2 Department of Biology, Indiana University, Bloomington, Indiana, United States of America; 3 Department of Physics and the Lewis-Sigler Institute for Integrative Genomics, Princeton University, Princeton, New Jersey, United States of America; 4 Plateforme de Microscopie, Institut de Microbiologie de la Méditerranée, Marseille, France; HHMI, Massachusetts Institute of Technology, United States of America

## Abstract

Eukaryotic cells utilize an arsenal of processive transport systems to deliver macromolecules to specific subcellular sites. In prokaryotes, such transport mechanisms have only been shown to mediate gliding motility, a form of microbial surface translocation. Here, we show that the motility function of the *Myxococcus xanthus* Agl-Glt machinery results from the recent specialization of a versatile class of bacterial transporters. Specifically, we demonstrate that the Agl motility motor is modular and dissociates from the rest of the gliding machinery (the Glt complex) to bind the newly expressed Nfs complex, a close Glt paralogue, during sporulation. Following this association, the Agl system transports Nfs proteins directionally around the spore surface. Since the main spore coat polymer is secreted at discrete sites around the spore surface, its transport by Agl-Nfs ensures its distribution around the spore. Thus, the Agl-Glt/Nfs machineries may constitute a novel class of directional bacterial surface transporters that can be diversified to specific tasks depending on the cognate cargo and machinery-specific accessories.

## Abstract 2

The *Myxococcus* Agl-Nfs machinery, a type of bacterial transport system, is modular and is seen to also rotate a carbohydrate polymer directionally at the spore surface to assist spore coat assembly.

## Author Summary

Many living cells use processive cytoskeletal motors to transport proteins and subcellular organelles to specific subcellular sites. In bacteria, this type of transport has yet to be identified and it is generally thought that random protein collisions underlie most biochemical processes. In recent years, our view of the bacterial cell was changed by the discovery of subcellular compartmentalization and a cytoskeleton, suggesting that processive motors might also operate in prokaryotes. We previously characterized a mechanism of intracellular transport that drives cell motility across solid surfaces in the gram-negative bacterium *Myxococcus xanthus*. Since the transport apparatus was also found in bacterial species that do not move on surfaces, we postulated that intracellular transport underlies other cellular processes in bacteria. Indeed, we show here that the *Myxococcus* motility motor can be adapted to transport sporulation-specific proteins around the nascent spore surface. Because the transported proteins are linked to the main spore coat, this motion assists the assembly of a protective spore coat. In conclusion, the *Myxococcus* motility/sporulation transport machinery defines an emerging class of versatile transport systems, suggesting that processive transport has been overlooked and may well orchestrate many processes in bacteria.

## Introduction

In eukaryotic cells, motor-assisted intracellular transport regulates fundamental cellular processes including cell division, macromolecule secretion, and cell migration. Because of its small size, the bacterial cell has long been considered to be a disordered compartment where biochemical reactions and cellular processes are governed by diffusion-driven random collisions. However, in recent years, it has become clear that bacteria are highly organized and contain a complex cytoskeleton [1],[2]. Despite the identification of bacterial counterparts of actin, tubulin, and intermediate filaments, processive cytoskeletal motors akin to myosin, kinesin, or dynein have yet to be found in bacteria [3]. Previously, while studying a mechanism of surface motility in *Myxococcus xanthus*, we have shown that the motility machinery consists of a new type of processive transport system (Agl-Glt) [4],[5]. Phylogenomic studies suggested that the motility function of the Agl-Glt machinery emerged from the recent specialization of an older system, predicting that bacterial Agl-Glt–like transporters may be adapted to other functions [5]. Here, we show that in the same bacterium, Agl, the motor component of Agl-Glt machinery, forms a second transport system that propels spore coat assembly proteins during sporulation. This finding suggests that a previously overseen type of bacterial surface transport can be adapted to mediate very different cellular tasks in prokaryotes.

*M. xanthus* cells move across solid surfaces by a process termed gliding (A)-motility where surface translocation occurs in the absence of extracellular organelles [6]. In recent years, remarkable progress has been made to elucidate the motility mechanism with the first identification of the motility machinery and the tracking of its localization in live gliding cells [7]. The motility machinery consists of a molecular motor, Agl (AglR, Q, and S), a three subunit flagellar-type proton channel that assembles in the bacterial inner membrane [4]. During motility, the Agl motor harvests the proton motive force (pmf) to move directionally along a looped continuous path spanning the entire cell length [8]. Helical trafficking of the motor may occur through a connection with the actin-like MreB cytoskeleton on the cytosolic side [8],[9]. In the cell envelope, mechanical work from the motor is transduced to the cell surface by the Glt (**Gl**iding **t**ransducer, GltA–K) complex through a direct interaction involving AglR and GltG [5],[10]. Transported Glt proteins produce thrust when the machinery comes in contact with the underlying substrate at areas termed focal adhesions (FAs) [4],[11]. How exactly the Glt proteins contact the substrate is unknown [7],[12], but adhesion is facilitated by slime, a yet undefined sugar polysaccharide specifically bound by the outermost components of the motility machinery [13]. Thus, the Agl-Glt machinery may be viewed as a modular transport system where an active transport unit (Agl) combines with a specific cargo (Glt) to propel the cells on surfaces.

**Figure 1:**
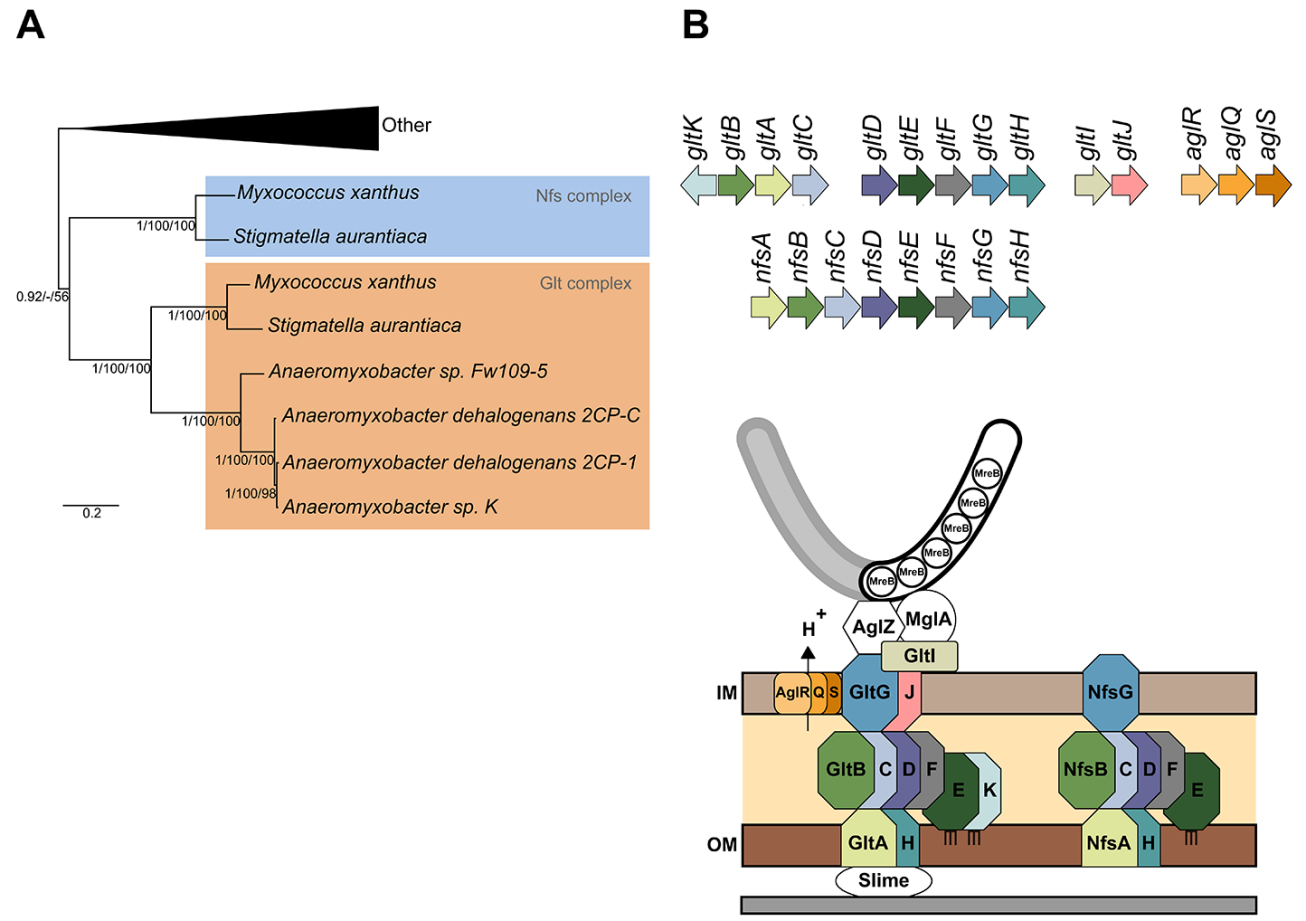
The Nfs complex is a Glt-transducer-like complex. (A) Phylogenetic relationship between the *nfs* and *glt* complexes inside the *Myxococcales* order. Shown is a rooted Bayesian phylogenetic tree of concatenated alignments of GltD, E, and G extracted from [5]. (B) Genetic organization of *agl*, *glt*, and *nfs* genes and predicted structures of the Agl-Glt and Nfs machineries from [5] and [13]. Paralogous genes are shown in specific colors. The PG is not represented because its connection to Glt and Nfs proteins is unknown.

Remarkably, the *glt* genes are paralogous to the *nfs* (necessary for sporulation, *nfsA–H*, Figure 1) genes involved in assembly of the *Myxococcus* spore coat [5],[14]. Bacterial sporulation is a stress-induced differentiation leading to the formation of highly resistant quiescent cell types. In the model bacteria *Bacillus* and *Streptomyces* sp., a spore is formed after elaboration of a complex proteinaceous outer shell, called the spore coat. However, the coat proteins and assembly mechanisms are unrelated (for reviews see [15],[16]). *Myxococcus* spores are formed following starvation in multicellular fruiting bodies. During multicellular development, the initiating signals have not been identified, but sporulation can be conveniently induced *in vitro* by the addition of glycerol [14]. Glycerol-induced spores display certain morphological differences compared to fruiting body spores (e.g., the absence of the outermost protein cuticula) [17], but they also share many similarities: resistance to heat and sonic disruption as well as germination. Contrary to *Bacillus* and *Streptomyces* spores, the *Myxococcus* spore coat is mostly composed of two carbohydrates, N-acetyl-galactosamine and glucose, in a molar ratio of 3∶1 [18],[19]. The exact structure of the spore coat polymer(s) is unknown, and glucose molecules may form an alpha 1,3-glycan chain independently from the N-acetyl-galactosamine [18],[19]. Recently, a locus of nine genes, named *exoA–I*, has been shown to be essential for spore coat synthesis [20]. Annotation of *exoA–I* indicates that the main spore coat polymer is likely a capsular-type polysaccharide, exported by an outer membrane Wza-like translocon (the ExoA protein) [20]. For simplicity, the *exo*-dependent spore coat polymer will be named Exo throughout the rest of this article. *Myxococcus* sporulation is a stepwise process. During the first step of sporulation, the peptidoglycan (PG) layer is seemingly degraded, an MreB-dependent process, which leads to cell rounding [20]. Subsequently, the Exo polymer is exported by ExoA and deposited around the collapsed inner and outer membranes [20]. Tight wrapping of Exo around the spore membranes requires the Nfs complex [20]. How exactly the Nfs system promotes spore coat assembly is unknown and is addressed in this article.

The Glt and Nfs proteins are highly similar and seem to associate with extracellular sugar polymers (slime and Exo, respectively). Thus, starting from the premise that both systems share similar operating principles, we investigated the function of Nfs in spore coat assembly. Doing so, we discovered that following the onset of sporulation, the Agl motility motor dissociates from the Glt complex and becomes recruited to the Nfs complex to transport it around the spore membranes. We further show that following its secretion at discrete sites around the spore surface, the Exo polymer is recruited by mobile Nfs units, suggesting that the Agl-Nfs machinery constructs the coat by wrapping Exo strands around the cell surface. We conclude that the Agl-Glt/Nfs machineries constitute a versatile class of active surface transport machineries that may carry out multiple functions in bacterial cell surface organization.

## Results

### The Nfs and Glt Complexes Are Paralogous Complexes

A recent phylogenetic study showed that the *glt* and *nfs* genes likely arose from the recent duplication of a single gene system in one of the terminal branches of the deltaproteobacteria (Figure 1A) [5]. Thus, the Nfs and Glt proteins are paralogues, and when predictable, protein domains are systematically conserved between Nfs and Glt proteins (Table S1). The *nfs* complex consists of eight genes, *nfsA–H* (Figure 1B), respectively, homologous to *gltA–H* (Table S1). Nfs is therefore predicted to be a somewhat simpler assemblage, missing paralogues to GltI, J, and K. Gene synteny is maintained between the *nfs* and *glt* clusters (Figure 1B). Of note, however, *gltA–C* and *gltD–H* form two distinct genomic clusters, but the *nfsA–H* genes are clustered in a single genomic region, possibly forming an operon (Figure 1B) [20]. Consistent with the notion that the *nfs* genes form a complex in the spore membrane, all *nfs* genes are essential for sporulation and their products localize to the cell envelope (tested for NfsA, B, C, D, E, and G and predicted for NfsF and H) [20], like their *glt* counterpart (tested for GltD, E, F, G, and H and predicted for GltA, B, C, J, and K) [5],[10]. Therefore, we predict that the Nfs proteins assemble a Glt-like membrane sporulation complex (Figure 1B).

### *aglRQS* and *nfs* Are Part of a Sporulation Pathway

**Figure 2:**
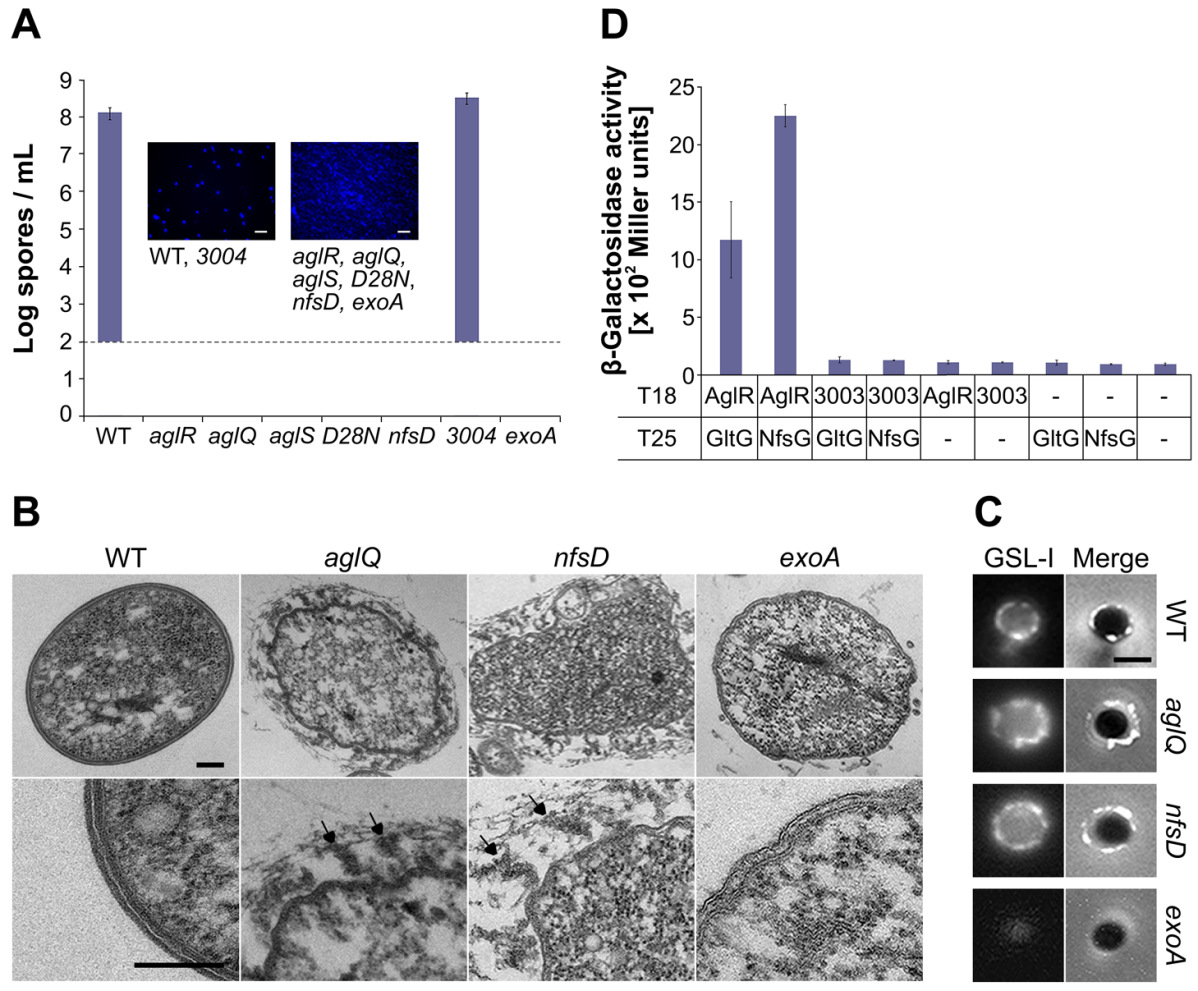
The Agl interacts with Nfs to promote spore coat assembly. (A) Sporulation titers after heat and sonication counted by DAPI staining in various strains. Corresponding DAPI-staining images are shown. Note the 10^2^ spores/ml detection limit of the assay. Scale bar = 5 µm. (B) Thin sections of myxospores observed by transmission electron microscopy. WT, *aglQ*, *nfsD*, and *exoA* strains were observed 24 h after the induction of sporulation. Arrows point to spore coat material that detaches from the surface of sporulating cells in *aglQ* and *nfsD* mutants. Scale bars = 0.1 µm. (C) GSLI-FITC staining of the spore coat material. WT, *aglQ*, *nfsD*, and *exoA* strains were observed 4 h after the induction of sporulation. Scale bar = 1 µm. (D) AglR interacts with GltG and NfsG in a bacterial two hybrid assay.

Previous works showed that Glt promotes motility in association with the Agl motor [4],[5],[8]. Therefore, although Nfs might be functional on its own, its function may also require an Agl-like motor. Reasoning that each complex may have its own dedicated motor, we tested whether the *MXAN_3003–5* genes, which encode the only additional complete set of Agl homologues in *M. xanthus* and are dispensable for motility, are required for sporulation like the *nfs* genes [5]. Because *nfs* mutants are defective for sporulation whether they are extracted from fruiting bodies or after glycerol-induction [14], we measured sporulation after glycerol induction throughout this study. Under these conditions, a mutant carrying an in-frame deletion in the *MXAN_3004* gene (the *aglQ* homologue) formed perfectly viable spores, indicating that the putative MXAN_3003–5 motor does not play a significant role in sporulation (Figure 2A).

We next tested whether AglRQS itself may be required for sporulation. Strikingly, the *aglR*, *aglQ*, and *aglS* mutants all showed a severe sporulation defect, comparable to the sporulation defects of the *nfsD* mutant and the *exoA* mutant lacking the Wza homologue (Figure 2A). An *aglQ_D28N_* point mutant, carrying a substitution in the AglRQS channel previously shown to abolish proton conductance and to paralyze the Agl-Glt motility machinery [4], also failed to sporulate (Figure 2A). Therefore, the AglRQS complex and, importantly, its proton-conducting activity are required for both motility and sporulation.

### *aglRQS* Are Required for Spore Coat Assembly

In the *nfs* mutants, the sporulation program is correctly initiated following glycerol induction: the cells round up and the Exo polymer is produced and exported to the cell surface [20]. However, in absence of Nfs, Exo is loosely attached to the spore surface, which results in loss of cell integrity and abortive sporulation [20]. By Transmission Electron Microscopy (TEM), we also observed a thin and complex electron dense layer around the membranes of WT spores [20], suggesting the presence of a spore coat (Figure 2B). In both the *nfsD* and *aglQ* mutants, this layer was absent and replaced by hair-like filaments that seemed loosely attached to the spore surface at one end (Figure 2B). Since the filaments were completely absent in an *exoA* mutant, they may constitute Exo polymer filaments (Figure 2B) [20]. To confirm that the filaments are not artifacts of TEM sections, we tested a fluorescein-coupled lectin, Griffonia (Bandeiraea) Simplicifolia lectin I (GSL-I), with a selectivity for *N*-acetylgalactosamine, the major sugar component of the spore coat [18],[21]. As expected, GSL-I stained the surface of WT spores but not that of spore-coat deficient *exoA* mutants (Figure 2C). On WT spores, GSL-I staining decorated the entire surface with occasional brighter dots (Figure 2C). Moreover, as expected if the spore coat was loosely attached, GSL-I staining of *aglQ* and *nfsD* mutants was not compact around the cell surface (Figures 2C and S1). Taken together, the TEM and lectin-staining experiments suggest that a function of the Agl-Nfs machinery is to promote the formation of a compact spore coat layer around the spore membrane. Additionally, we identified GSL-I staining as a useful tool to detect the spore coat by live fluorescence microscopy (see below).

### AglRQS Associates with Nfs to Form a Sporulation-Specific Machinery

In the gliding machinery, AglRQS contacts the Glt complex through the specific association of AglR and GltG (Figure 2D) [5]. We used a bacterial two-hybrid assay to test whether AglR also contacts the Nfs system through an interaction with NfsG, the GltG paralogue. Consistent with interaction, a significant β-galactosidase activity (>1,000 Miller units) was obtained when AglR and NfsG were expressed together (Figure 2D). No significant β-galactosidase activity was measured when each protein was expressed alone or when MXAN_3003 (the AglR homologue) was co-expressed with NfsG or GltG (Figure 2D). Thus, the AglRQS motor can associate with both the Glt and Nfs complexes.

### A Genetic Switch Activates the Agl-Nfs Machinery at the Onset of Sporulation

**Figure 3:**
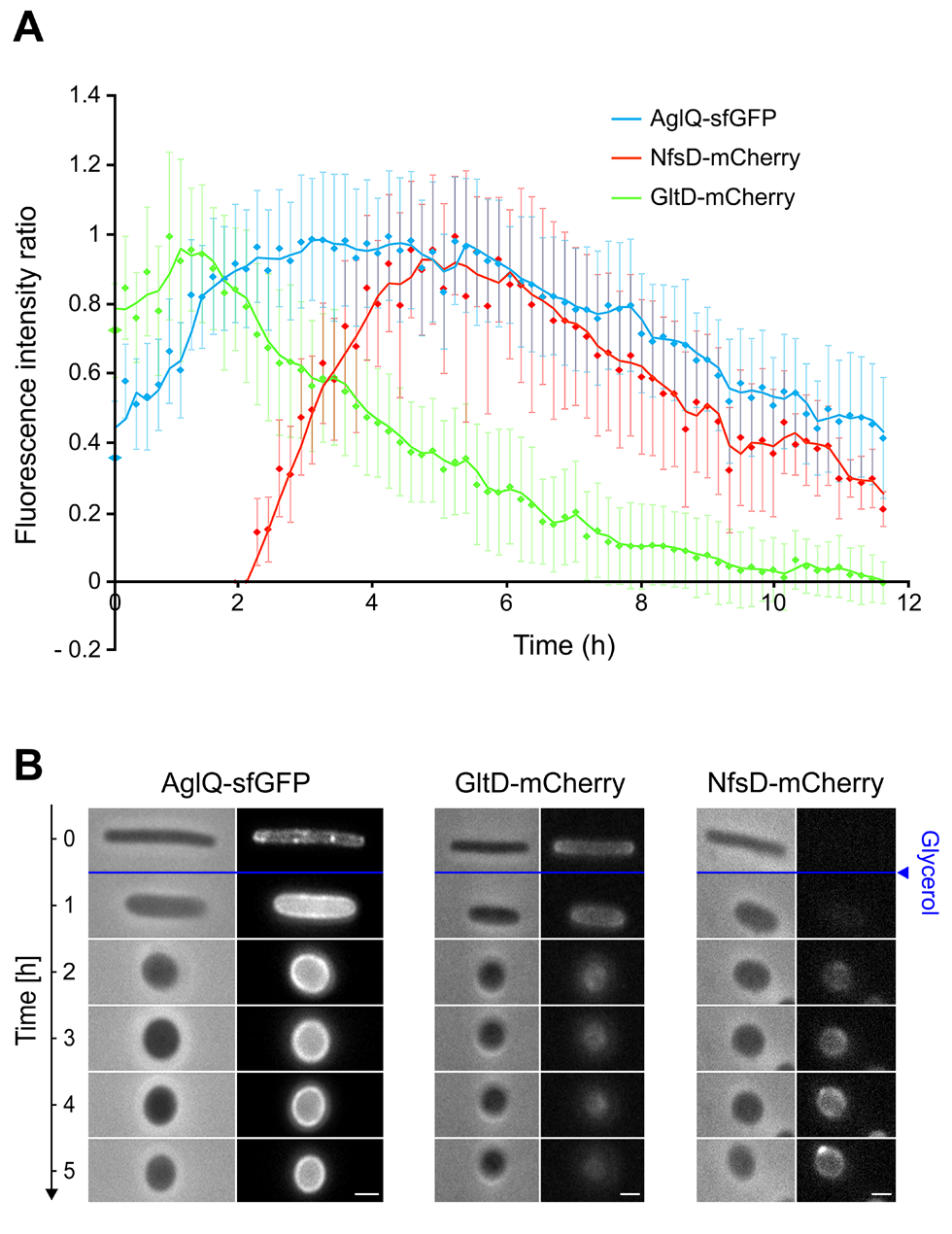
The Agl-Nfs machinery is sporulation-specific. (A) *nfs* and *glt* genes are differentially regulated during sporulation. Expression of NfsD, GltD, and AglQ as inferred from the measurements of relative single cell mCherry/sfGFP fluorescence intensities over time after sporulation induction. Shown are the average fluorescence intensity ratios (measured intensity/maximum intensity) of 25 cells for each time points. (B) Time course of AglQ-sfGFP (left), GltD-mCherry (middle panel), and NfsD-mCherry (right panel) dynamics during sporulation.

*Myxococcus* cells do not complete the sporulation process when spotted directly on an agar pad atop a microscope slide. Thus, to monitor sporulation by live microscopy and elucidate how the Agl-Nfs machinery drives spore coat assembly, we developed a microfluidic chamber assay where cells are immobilized and sporulate in liquid directly on the microscope stage (Figure S2A,B and Movie S1). Under these conditions, viable spores were obtained approximately 250–300 min after glycerol addition (Figure S2A,B). To test the dynamics of the Agl-Nfs machinery during sporulation, we constructed functional NfsD-mCherry and AglQ-sfGFP (super-folder GFP) [22] fusions to use as proxies to monitor both Nfs and Agl dynamics (Figure S3). Time-course sporulation experiments showed that NfsD-mCherry is expressed in lieu of GltD-mCherry (the motility NfsD paralogue) during sporulation, suggesting that sporulation depends on a genetic switch that results in the substitution of the Glt complex by the Nfs complex (Figures 3A–B and S4). Consistent with its role in spore coat assembly, expression of AglQ was even increased up to two-fold at the onset of sporulation and maintained at high level throughout the sporulation process (Figure 3A–B).

### The AglRQS Motor Rotates the Nfs Complex Around the Spore Surface

**Figure 4:**
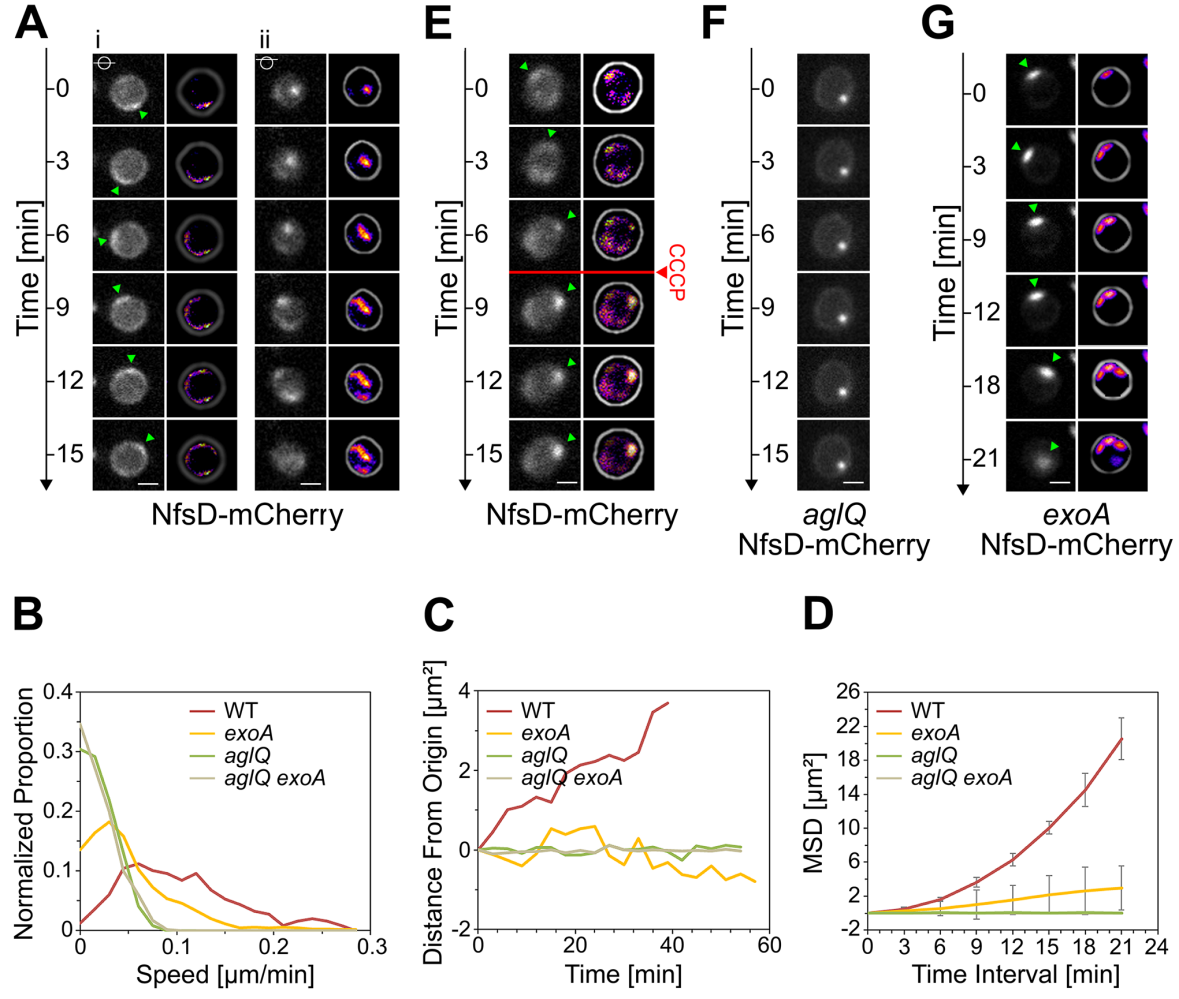
The AglRQS motor rotates the Nfs complex around the spore surface. (A) NfsD-mCherry moves in orbital trajectories around the surface of 4-h-old spores. Observation of dynamic NfsD-mCherry clusters at different focal planes, middle (i) and top (ii) sections of a spore. For each time lapse, trajectories were computed by summing the different time points in consecutive frames and shown in “fire” colors to the right. Scale bar = 1 µm. (B) Instantaneous speed histogram of tracked NfsD-mCherry clusters over time in WT spores and in the different mutants. (C) Distance from the origin of NfsD-mCherry clusters in WT spores and different mutants. (D) Mean square displacement (MSD) of NfsD-mCherry clusters over time in WT spores and in the different mutants. (E) NfsD-mCherry rotation is abolished by CCCP. (F) Rotation of NfsD-mCherry requires AglQ. (G) Movement of NfsD-mCherry in the *exoA* mutant.

In sharp contrast to the focal localization of AglQ during motility [4], AglQ-sfGFP accumulated circumferentially all around the spore membrane throughout the sporulation process (Figure 3B). However, when we analyzed the localization of NfsD-mCherry, we found that this protein covered the entire cell surface during the initial stage of sporulation but later formed fluorescent-bright clusters at the end of the cell-rounding phase, a sporulation stage where the Nfs complex would be expected to become active (Figure 3B). In time-lapse experiments, the NfsD-mCherry foci were observed to move in orbital trajectories around the spore surface (Figure 4A and Movie S2). Orbital trajectories could be captured both when the microscope focal plane was set to the middle or to the top of a sporulating cell, showing that NfsD rotates all around the spore circumference (Figure 4Ai and 4Aii). Rotating NfsD-mCherry clusters could be tracked for distances between 3/4 and up to a full spore circumference, suggesting a high level of directionality (Figures 4Ai and S5). To measure the movement parameters of NfsD-mCherry precisely, we designed a computational method to measure the distance traveled by NfsD-mCherry foci at subpixel resolution. Moreover, since sporulating cells are spherical, the distance that separates two clusters at distinct times is not the euclidian distance but the orthodromic distance (Figure S5). Orthodromic distances traveled by NfsD-mCherry clusters were thus calculated for each time point by projection (see Methods and Figure S5). This analysis revealed that NfsD-mCherry foci moved at instantaneous speeds ranging from 0.1–0.3 µm.min^−1^ (Figure 4B). Movement was clearly directional because (i) the distance to the origin increased over the time (Figure 4C) and (ii) NfsD-mCherry movements could be described by a Mean Square Displacement (MSD) over time that increased by 4*Dt+v^2^t^2^* relation (with *v* the mean velocity, *v* = 0.12±0.05 µm.min^−1^, and *D* the apparent diffusion coefficient, *D* = 0.012±0.007 µm^2^.min^−1^, Figure 4D). This directional movement must be linked to the activity of the Agl motor because movement was immediately stopped by addition of Carbonyl Cyanide-m-ChloroPhenylhydrazone (CCCP), a proton motive force uncoupler (Figures 4E and S7C and Movie S3) and paralyzed NfsD-mCherry foci formed both in the *aglQ* mutant and in the *aglQ_D28N_* mutant (Figure 4B,C,F and unpublished data). MSD analysis of NfsD-mCherry clusters in the *aglQ* mutant showed a typical subdiffusive behavior, consistent with the absence of significant active movements (Figure 4D).

### The Agl-Nfs Machinery Transports Glycan Strands Across the Surface of Sporulating Cells

**Figure 5:**
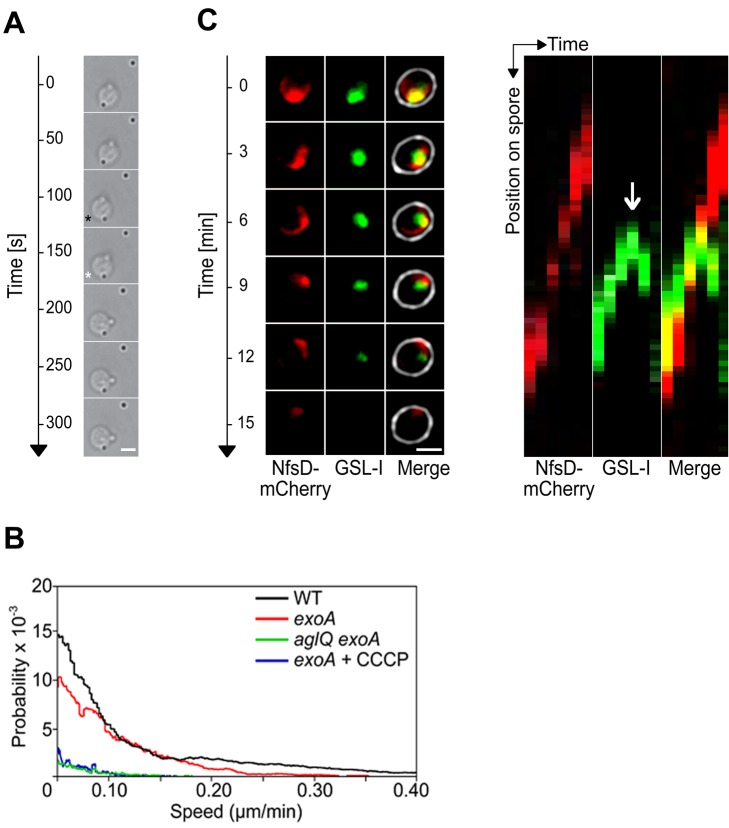
The Nfs complex transports the spore coat polymer at the surface of the developing spores. (A) Snapshots of two beads moving on spore surface. Two beads are attached to a WT spore; another bead is stuck to the bottom of flow chamber providing a fixed reference. The bead in focus is tracked using the low-powered laser as in the Methods section. Both beads move independently at different times (arrows). Scale bar = 1 µm. (B) Speed histogram of tracked polystyrene beads at the spore surface: WT spores, *exoA* spores, *exoA* spores in the presence of CCCP (10 µM), and *exoA aglQ* spores. WT spores in the presence of CCCP and *aglQ* spores yielded similar results as the *exoA* CCCP and *exoA aqlQ* spores and are therefore not represented for improved clarity. (C) Dynamics of NfsD-mCherry and GSLI-FITC on a sporulating cell. A time lapse of a 4-h-old sporulating cell is shown with its corresponding kymograph. The white arrow points to the dissociation of the red and green signals. Scale bar = 1 µm.

During motility, Agl functions to transport Glt proteins and slime along the cell surface [4],[11]. Thus, the Agl motor may transport the Nfs complex and associated Exo strands to construct a densely packed spore coat around the spore membrane. We used a micron-sized polystyrene beads assay [4] to test directly whether Agl has a surface transport activity and found that beads were transported along the spore surface with instantaneous speeds matching the dynamics of NfsD-mCherry (0.1–0.3 µm.min^−1^, Figure 5A–B). Consistent with bead transport by the Agl motor, this transport was undetectable in the *aglQ* mutant and abolished by the addition of CCCP (unpublished data and Figure 5B).

To test whether Exo is the terminal cargo of the Agl-Nfs machinery, we stained NsfD-mCherry–expressing spores with GSL-I and imaged each fluorophore simultaneously 4 h after the induction of sporulation. As already mentioned, GSL-I staining covers the entire spore surface but also forms prominent fluorescent clusters (Figure 2C). When these clusters were imaged by time-lapse, they rotated together with NfsD-mCherry clusters (Figure 5C and Movie S4). Co-tracking of NfsD-mCherry and GSL-I foci were occasionally observed to dissociate, which was followed by rapid dispersal of the GSL-I cluster (Figure 5C). Thus, Exo-linked rotating Agl-Nfs complexes are likely involved in the construction of a densely packed protective mesh at the surface of developing spores.

### Nfs Transport Does Not Require the MreB Cytoskeleton

Mature spores apparently lack PG and the actin-like MreB cytoskeleton [20],[23]. Consistent with published data [20], we found that MreB is only required for the initial cell rounding phase of sporulation using A22, a specific MreB-inhibitor (Figure S6A–B) [9]. Since the activity of the Agl-Glt motility machinery requires MreB [8],[9],[11], we tested the effect of A22 on NfsD-mCherry dynamics and found that MreB is dispensable for NfsD-mCherry rotation (Figure S6C). Contrarily to *Bacillus* endospores, myxospores do not contain PG or only trace amounts [23]. The Exo spore coat may provide spore integrity in absence of other rigid cellular scaffolds because *exo*, *nfs*, and *agl* mutants all show aberrant cell morphologies after 24 h (Figure S2A) [20]. Thus, during sporulation, both MreB and the PG seem dispensable for the activity of Agl-Nfs.

### The Exo Polymer Is Essential for Transport Directionality

What is the mechanism of Nfs transport? In absence of a rigid scaffold (i.e., MreB filaments and the PG), Agl motor units may distribute circumferentially (Figure 3B) to transport Nfs proteins from one motor unit to the next, similar to actin filaments being moved by immobilized Myosin motors [24]. Alternatively and because the Nfs proteins are terminally associated with the Exo polymer, Exo secretion itself could push Nfs proteins around the spore surface in a mechanism reminiscent of PG glycan strand insertion rotating PG synthetic complexes [25]–[27]. To discriminate between these two possibilities, we tested NfsD-mCherry dynamics in the *exoA* mutant. In the absence of ExoA, several critical features of Nfs transport emerged: (i) NfsD-mCherry clusters formed foci that appeared smaller in *exoA* mutant than in WT cells, suggesting that Exo polymers organize NfsD-mCherry clustering at the spore surface (compare Figure 4G and A); and (ii) NfsD-mCherry movement was erratic and characterized by frequent reversals and saltatory motions, suggesting that directionality is lost in the mutant (Figure 4G). To test this possibility, we computed the MSD of NfsD-mCherry clusters as a function of time in *exoA* mutant cells and found it to be mostly linear, a characteristic of undirected random motion (Figure 4D). In contrast, the MSD of WT cells is characteristic of directed motion (Figure 4D).

In the *exoA* mutant, although NfsD-mCherry clusters appear to move randomly, this movement is unlikely to be driven by diffusion alone because many clusters showed short and fast movement phases with burst speeds similar to NfsD-mCherry clusters in WT cells (Figure 4B,C,G). These fast movements depend on the activity of the Agl-motor because (i) they were completely abolished by CCCP (Figure S7A) or in an *exoA aglQ* double mutant (Figures 4B,C,D,G and S7B) and (ii) the MSD of NfsD-mCherry was constant over time in the *exoA aglQ* double mutant (Figure 4D). Finally to prove that AglQ-dependent transport can still occur in absence of the Exo polymer, we further tested bead transport in absence in the *exoA* mutant. Similar to the movements of NfsD-mCherry clusters in the *exoA* mutant, bursts of fast bead movements were observed in this strain (Figure 5B). Since movements were completely abolished by the addition of CCCP or in an aglQ mutant background and thus depend on the activity of the Agl motor (Figure 5B), all together, these results show that surface movements at the cell surface (Nfs and beads) result from active Agl-dependent transport and not spore coat polymer secretion, which may instead serve to guide motion (see Discussion).

### The Exo Export System Localizes at Discrete Sites Around the Spore

**Figure 6:**
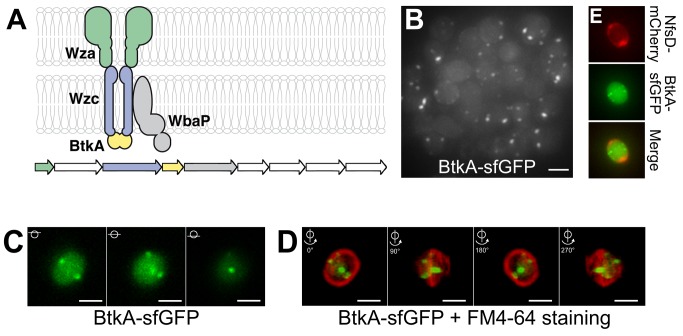
The Exo export system localizes at discrete sites around the spore surface. (A) Predicted structure of the Exo export apparatus and genetic organization of the *exoA–I* operon. White genes have no know homologs in the databases. Gene accession numbers (MXAN) are shown. (B) Projection of a Z-stack of 4-h sporulating cells and associated BtkA-sfGFP foci. Scale bar = 1 µm. (C) Z-sections of a sporulating cell and localization of BtkA-sfGFP (D) Snapshots of a 3D reconstruction of a cell expressing BtkA-sfGFP. The spore membrane was stained with FM4-64. 90° rotations are shown. Scale bar = 1 µm. (E) BtkA-sfGFP foci do not colocalize with NfsD-mCherry foci. Shown are 3D projections of z-sections for BtkA-sfGFP, NfsD-mCherry foci and the corresponding merge. For each probe, the individual z-sections are shown in Figure S8. Scale bar = 1 µm.

Gene homologies suggest that the *exoA–I* genes responsible for spore coat synthesis encode a capsular polysaccharide synthesis and Wza-type export apparatus (Figure 6A) [20]. To further understand the link between Agl-Nfs activity and the export of Exo to the spore surface, we localized structural components of the export apparatus. Fusions to ExoA and ExoC, respectively, encoding Wza and transmembrane Wzc-domain homologues were nonfunctional (unpublished data). Although in general proteobacterial Wzc proteins carry both a transmembrane and a cytosolic BY-kinase domains, in *Myxococcus* the Wzc transmembrane domain and the kinase Wzc domain are carried by two distinct polypeptides (respectively named ExoC and ExoD/BtkA) [28], a conformation often found in firmicutes [29]. Nevertheless, BtkA-dependent tyrosine phosphorylation of the Wzc-like transmembrane polypeptide is essential for *Myxococcus* sporulation [28], suggesting that BtkA can be used to localize Exo export sites. We successfully obtained a functional BtkA-sfGFP fusion (Figure S3). Consistent with previous expression studies [28], BtkA-sfGFP was only expressed following sporulation induction. At 4 h, BtkA-sfGFP formed prominent foci in ≈50% of the cells (Figure 6B–C). Each cell contained on average 1.7±1 clusters (counted for 305 cells, with a maximum number of clusters of six per cell), all located near the spore membrane suggesting that BtkA-sfGFP is indeed recruited to the export apparatus. Z-sections of 4-h-old spores and 3D reconstructions further revealed that BtkA-sfGFP foci form at discrete sites around the spore periphery (Figure 6D and Movie S5). Importantly, the BtkA-sfGFP foci did not colocalize with NfsD-mCherry and often formed in distinct z-planes around the spore (Figures 6E and S8). In total the BtkA localization data suggest that sporulating cells only assemble a few discrete fixed export sites in the cell envelope, suggesting that rotating Agl-Nfs machineries act downstream from Exo secretion to construct the spore coat (see Discussion).

## Discussion

### Agl-Glt/Nfs Machineries Are Surface Transport Systems

**Figure 7:**
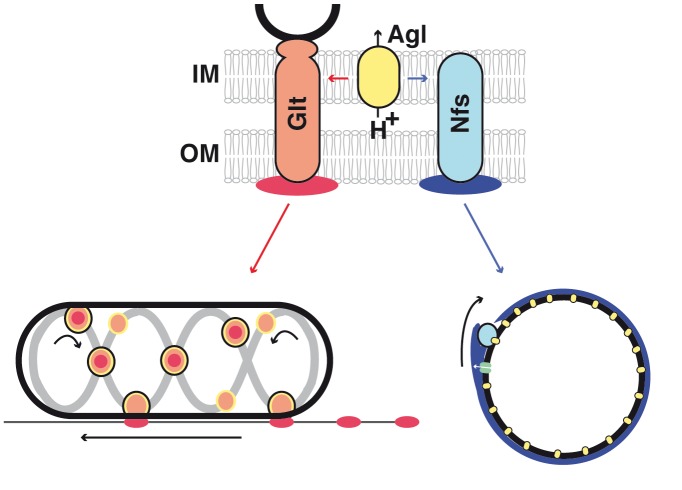
Phase specific interactions between Agl and Glt/Nfs promote motility or sporulation. The Agl motor (yellow), a three protein MotAB-like channel harvests the pmf and interacts either with Glt (orange) or Nfs (light blue) to transport slime (red) or the Exo polymer (blue) depending on the growth phase. Both Glt and Nfs are shown spanning the entire cell surface because they both contain predicted inner and outer membrane proteins. The connection between the Glt complex and the MreB cytoskeleton is shown in black. IM, inner membrane; OM, outer membrane; the PG is not represented because its connection with Glt is unresolved and spores apparently lack PG. In both cases, the transport mechanism remains to be elucidated. In motile cells, the current model proposes that Glt proteins and attached slime (orange-red dots circled in black) traffic along a closed loop helix (grey) and generate propulsion as they interact with the underlying substratum. At the lagging cell pole, the motility complexes become de-activated (orange circles), potentially by removing their connection to slime, which thus becomes deposited on the substrate. In spores, Nfs proteins may associate with Exo polymers following their secretion by the Exo export machinery (green). Distributed Agl motor units could move Exo-linked Nfs complexes from one motor to the next, guided by the Exo polymer.

In this report, we show that the Agl motility motor is modular and interacts with Glt or Nfs proteins, depending on the growth phase. The output of these interactions is remarkably different because Agl-Glt drives gliding motility while Agl-Nfs drives spore coat assembly (Figure 7). Both Agl-Glt and Agl-Nfs interact with extracellular polysaccharides (respectively, slime and the Exo polymer) and the specific function of each system may be linked, at least partially, to the chemical properties of the cognate polymer. For example, motility could be facilitated by the chemical adhesiveness of slime, while the structure of Exo might make it particularly suited to form a coat around the spore membrane (Figure 7).

Since both slime and the Exo polymer are still detected at the cell surface in *agl* mutants, Agl-Glt/Nfs systems are not involved in the synthesis/export of associated sugars. This is particularly clear during sporulation where the synthesis and transport of the Exo polymer is controlled by the *exo* locus. Rather, several lines of evidence suggest that Agl-Glt/Nfs system transport their cognate polymers after export along the cell surface: (i) AglRQS form a flagellar motor-like complex, a predicted pmf-driven motor that interacts both with Glt and Nfs proteins (through a specific interaction between AglR and GltG/NfsG). Accordingly, both the Agl system and the pmf are required for Glt and Nfs movements (this work and [8],[11],[30]). (ii) Agl exerts transport activity at the surface of motile cells and spores, monitored by addition of polystyrene beads. Transport of slime and Exo was observed directly with fluorescent lectins, and in both cases mobile lectin patches were translocated together with mobile AglQ (slime) and NfsD (Exo) complexes (this work and [13]). (iii) During sporulation, secretion of the Exo polymer is not a significant driving force for Nfs movement. Thus, rotation does not result from a pushing action of the Exo polymer, for example, like when MreB-associated PG synthetic complexes are moved circumferentially by the incorporation of new glycan strands in the PG meshwork [25]–[27]. Importantly, however, transport directionality was lost in absence of Exo polymer secretion. When Agl-Nfs–linked Exo strands become deposited at the spore surface, they could act like a molecular ratchet, preventing motor back steps and restricting Agl-Nfs movements in one dimension. The rigidity of the growing Exo meshwork may also support Nfs movements, especially since MreB and the PG may not be involved or present. In the motility system, slime could perform analogous functions and explain the mysterious directionality of Agl-Glt complexes. Unfortunately this could not be tested because the main slime polymer has not been characterized.

Although it is still unclear how the Agl-Glt machinery accommodates the rigid PG layer during gliding, the situation may be simpler in spores: circumferential AglQ motors may transport Nfs subunits from one motor to the next, fueled by the pmf and guided by linked Exo strands. Testing this mechanism further will require defining the structure of the Exo polymer and its connection with the Nfs proteins.

### Spore Coat Assembly by Agl-Nfs

The connection between Agl-Nfs and Exo and the severe spore coat assembly defect observed in *nfs* and *agl* mutants suggests that Agl-Nfs mediates spore coat assembly directly. As discussed above Nfs function can be separated from secretion. Using a BtkA-sfGFP fusion, we localized the Exo secretion apparatus and found it to form a limited number of sites around the spore periphery, suggesting that secretion is spatially constricted. This localization is not surprising because immunolocalization of Wza only suggested that the export apparatus forms discrete sites in *Escherichia coli* [31]. Thus, a rotary transport complex may be needed to build a homogenous glycan layer all around the spore. Our experiments and others [20] suggest that the activity of Agl-Nfs is necessary to anchor the spore coat polymer at the surface. In this process, scanning Agl-Nfs complexes may capture newly secreted glycan Exo strands and incorporate/deposit them where necessary in the growing spore coat meshwork. This could occur, for example, if a glycosyl transferase activity is linked to the Nfs complex. However, other mechanisms are also possible and more work is needed to understand how Agl-Nfs contributes to spore coat assembly at the molecular level.

### Agl-Glt/Nfs Proteins Define a Versatile Class of Transport Systems in Bacteria

The Agl-Glt/Nfs machineries likely evolved by modular expansion of a conserved system of seven proteins (Figure S9) [5]. This “core” system is found as a standalone machinery in many gammaproteobacteria and some deltaproteobacteria, suggesting that it carries function [5]. Because the core complex consists of an Agl-like motor and a simplified Nfs/Glt-like apparatus (Figure S9), this machinery may constitute a basal transport machinery. A survey of Agl-Glt/Nfs-like machineries in the deltaproteobacteria shows that these machineries adopt many potential conformations and therefore may cover a potentially broad functional repertoire (Figure S9) [5]. Functional specialization of Agl-Glt/Nfs machineries may be linked to the type of transported cargo, and thus expansion of the core system in the deltaproteobacteria may have evolved diverse tasks, sugar polymer transport (Nfs and Glt), but also potentially protein and lipid transport. Even though *glt* and *nfs* paralogues have a rather narrow distribution [5], the Agl motor itself is a member of a ubiquitous family of bacterial motors (Mot/Tol/Exb) [4]. Therefore, it is conceivable that other motor associations also promote processive transport in bacteria.

Many Agl-Glt/Nfs systems are present in bacteria that are not currently genetically tractable. Computational genetic reconstruction of ancestral assemblages is an emerging powerful means to explore both the function of a macromolecular complex and how it evolved [32]. In future works, the reductive genetic study of the Agl-Glt/Nfs machineries in *Myxococcus* should be instrumental to characterize functional intermediates and test the functional repertoire of these systems. Such study would also allow deciphering the evolution of this complex biological machine with high likelihood, an emerging challenge in evolution biology [33]. Finally, critical traits of the *Myxococcus* lifestyle evolved from the diversification of a single molecular system, showing that modifications of a single genetic system can give rise to profound ecological adaptations.

## Materials and Methods

### Bioinformatics Analysis of *glt* and *nfs* Genes

Protein sequences were analyzed by BlastP (NCBI) searches of the nonredundant (nr) and Pfam (release 24.0) (see comment on Table S1) databases [34]. Signal peptides signal and transmembrane helices were the predicted using the signalP 3.0 [35] and TMHMM v.2.0 [36] servers, respectively.

### Bacterial Strains, Plasmids, and Growth

Strains, primers, and plasmids are listed in Tables S2, S3, and S4. See Tables S2, S3, and S4 for strains and their mode of construction. *M. xanthus* strains were grown at 32°C in CYE rich media as previously described [37]. Plasmids were introduced in *M. xanthus* by electroporation. Mutants and transformants were obtained by homologous recombination based on a previously reported method [37]. *E. coli* cells were grown under standard laboratory conditions in Luria-Bertani broth supplemented with antibiotics, if necessary.

### Bacterial Two-Hybrid Experiments

Bacterial two-hybrid experiments, plate, and β-Galactosidase assays were performed as previously described [5] and as recommended by the manufacturer's instructions (Euromedex).

### Determination of Spore Titers

Sporulation was induced by adding glycerol to a final concentration of 0.5 M directly to a flask of CYE-grown *Myxococcus* cells (OD_600_ of 0.5) as previously described [14]. Viable spore titers were determined after 24 h based on resistance to heat and sonication. For this, 10 mL cells were harvested, pelleted at 5,000×*g* during 5 min at room temperature, resuspended in 10 ml sterile water, incubated at 50°C for 2 h, and sonicated three times (30 pulses, output 3, 50% duty) in ice water. The surviving spores were counted directly on the microscope after 10 min incubation in a DAPI staining solution (formaldehyde 4%—DAPI 1 µg/mL) with a detection limit of 10^2^ spores/ml.

### Western Blotting and Spore Protein Extraction

Protein lysates for Western blot analysis were generated by harvesting sporulating cells. Spores were pelleted and resuspended in Tris-HCl 50 mM pH 7.8 supplemented with benzonase (Sigma) and phenylmethanesulfonylfluoride (PMSF, Sigma). Spores were lysed in a Fast-Prep-24 at 6.5 m.s^−1^ for 45 s, 8 times (matrix lysing B, MP Biomedicals, France). Protein concentrations were determined by Bradford assay (Bio-Rad) according to the manufacturer's instructions. Lysates were solubilized in Laemmli sample buffer and resolved by sodium-dodecyl sulfate polyacrylamide gel electrophoresis (SDS-PAGE) using 8% polyacrylamide concentration for GltD. Proteins were transferred to nitrocellulose membrane (0.45 µm, Bio-Rad) using a tank transfer system (Bio-Rad) and probed with anti-GltD rabbit polyclonal antibody [5] at a 1∶1,000 dilution and HRP-conjugated goat anti-rabbit secondary antibodies (Bio-Rad) at 1∶5,000. The signals were revealed with a SuperSignal West Pico Chemiluminescent Substrate (Thermo Scientific) and imaged in a LAS4000 Luminescent Image Analyzer (GE Healthcare).

### Live Microscopy Sporulation Assay

Commercial microscopy chambers (Ibitreat uncoated, Biovalley) were filled with 100 µL of carboxymethylcellulose sodium salt (medium viscosity, Sigma-Aldrich) solution (1.5 mg/mL) diluted in de-ionized water and left 15 min at room temperature. The excess of coating solution was removed by flushing with de-ionized water first, followed by flushing with TPM buffer (10 mM Tris pH 7.6; 8 mM MgSO_4_; 1 mM KH_2_PO_4_). The channels were then filled with 100 µL of a CYE-grown cell suspension (OD_600_ = 0.5) previously washed and resuspended in TPM solution and left at room temperature during 20 min. Nonadhering cells were flushed with TPM, and sporulation was induced by adding glycerol to a final concentration of 0.5 M directly on the microscope stage. Time-lapse experiments and image processing were performed as previously described using an inverted Nikon TE2000-E-PFS inverted epifluorescence microscope [38].

All image analysis was performed under Image J (NIH). Fluorescence quantifications were performed by integrating fluorescence intensities and normalizing over the level of background fluorescence measured for cells that do not express fluorescent proteins. Kymographs were obtained as follows: a typical NfsD-mCherry cluster path was obtained by summing a stack of time-lapse images. Kymographs were then computed by reslicing along that path, defined with the “Segmented Line” selection tool.

To measure the fluorescence areas and discriminate GSL-I staining in various mutants, we first defined the specific signal by thresholding and removing background fluorescence, by subtraction. For each cell, the total area of GSLI-specific fluorescence was measured and normalized by the total area of the cell. Box plots were then computed under R.

In drug experiments, Carbonyl Cyanine-M-Chlorophenylhydrazone (CCCP, 1 mM, Sigma Aldrich) and A22 (50 µg/ml, Calbiochem) were injected manually into the flow chamber. For lectin staining, a Griffonia (Bandeiraea) simplicifolia (GSL)-FITC conjugated 2 mg/mL stock solution (CliniSciences) was diluted 1∶100 in TPM containing 1 mM of CaCl_2_ and 100 µg/mL of bovine serum albumin (BSA, Sigma-Aldrich) immediately prior to injection. The mixture was then injected. The unbound lectin was washed (TPM, CaCl_2_ 1 mM, BSA 100 µg/mL) out of the flow chamber after 40 min of incubation.

### Subpixel Resolution Tracking of Fluorescent Foci at the Surface of Sporulating Cells

For each experiment, stacks of images were first normalized to correct for background fluctuations over time. If required, the background intensity of phase contrast images was subtracted to optimize auto-thresholding operations. Cell boundary and major and minor axis were detected using a specifically developed plug-in for ImageJ. Briefly, cells were detected using an autothresholding function, and subpixel resolution refined cell contours were obtained using a cubic spline-fitting algorithm. Major axis was deduced from the skeleton and expended in both directions to the most probable point, maximizing the cell-boundary curvature and minimizing the angle between the centerline and the cell boundary. Fluorescent foci were detected using a local and subpixel resolution maxima detection algorithm. The position of each focus is first determined using a polar coordinate system (r, θ), where the radial distance (r) represents the distance from the focus position and the cell center and the angular coordinate (θ) represents the angle formed between the major cell axis and the focus (Figure S5). Since sporulating cells are spherical and NsfD-mCherry foci form close to the cell surface, the polar coordinate system was extended to a 3D spherical coordinate system (p, θ, φ), where the radial distance (p) represents the distance from the cell center to the cell boundary, the angular coordinate (θ) represents the angle formed between the major cell axis and the orthogonal projection of the focus position on the focus plane, and the azimuthal angle (φ) is deduced using the relation φ = arcos(r/p) (Figure S5). The distance between two points at the surface of a spherical spore of respective spherical coordinates (p1, θ1, φ1) and (p2, θ2, φ2) was deduced from the relation d = p·arcos(cos(θ1) ·cos(φ1) · cos(θ2) · cos(φ2)+sin(θ1) · cos(φ1) · sin(θ2) · cos(φ2)+sin(φ1) · sin(φ2)). By convention, a positive angular coordinate means that the angle θ is measured counterclockwise from the polar axis formed by the major axis.

Fluorescent foci were tracked over time with a specifically developed plug-in for ImageJ. Briefly, cells are tracked with an optimized nearest-neighbor linking algorithm using the polar (r, θ) or the spherical coordinates (p, θ, φ). From foci trajectories, the distance between two temporal points was used to calculate instantaneous speeds shown in Figure 4B and combined with the cumulated distance and the distance from the origin to compute the Mean Square Displacement (MSD). For WT cells, the mean velocity and the diffusion coefficient were extracted from the second order fit of the MSD. For each condition tested, the MSD of at least 15 individual foci trajectory was calculated.

### TEM

*M. xanthus* cells were fixed for 1 h with glutaraldehyde 2.5% in CYE medium and postfixed 1 h in 2% OsO_4_. Then cells were dehydrated in ethanol and embedded in epon. Ultrathin sections were stained with aqueous uranyl acetate for 10 min and lead citrate for 5 min. Sample observations were performed on a Tecnai-G2 LaB6 microscope (FEI Company) operating at 200 kV.

### Bead Tracking

Custom microscopy chambers were made of a 1 mm-thick coverslide and a thin coverslip (#1, thickness 100 µm) separated by a double layer of double-sided sticky tape (Scotch). Chambers were immersed using 1.5%-agarose in DMSO (6 M, Sigma-Aldrich) for 15 min. Then, chambers were extensively washed with TPM containing 10 mM glucose. The chambers were filled with CYE-grown cell suspension (OD_600_ = 0.8) exposed to glycerol (final concentration of 0.5 M) 2 h or 4 h prior to experiments, and washed and resuspended in TPM solution directly before infusion. After 30 min, access was washed out, leaving sporulated and nonsporulated *M. xanthus* stuck to the surfaces of the microscopy chamber. Submicron-sized polystyrene beads (diameter 520 nm) were gently placed atop the sporulating cells using an optical trap system as previously described [4]. A low-powered tracking laser (<1 mW power at the sample, wavelength 855 nm) was focused on a spore-attached bead. The forward scattered laser light was collected on a position-sensitive photodiode (Model 2931, New Focus). The bead position was recorded and used to update the stage position using a PID feedback at a frequency of 50 Hz. The accuracy of this technique was measured to be better than 4 nm. Simultaneously, we used an EMCCD camera (iXon, Andor) to record time-lapsed videos at 1 Hz [4]. Video and high-resolution tracking data were recorded for several hours for all experimental conditions.

## Supporting Information

Figure S1 **Loose anchoring of the main spore coat polymer in the** ***aglQ*** **mutant.** Box plot representations of the ratio between the area of GSL-I fluorescence and a cell total area (fluorescence area ratio) are shown. For each strain, measurements were performed over 20 cells.
(TIF)

Figure S2 **Sporulation of** ***Myxococcus*** **cells in the microfluidic chamber.** (A) Sporulation kinetics after addition of glycerol. Following induction, cell rounding is observed with kinetics similar to cell rounding in liquid flasks. Aberrant cell shapes are observed both with the *aglQ* and *nfsD* mutants as described by [20]. Scale bar = 1 µm. (B) Germination after CYE (rich) medium injection. Spores germinate indicating that the observed round cells are indeed spores and not spheroplasts.
(TIF)

Figure S3 **AglQ-sfGFP, NfsD-mCherry, and BtkA-sfGFP are fully functional for sporulation.** Spore titers were determined and expressed as in Figure 2A.
(TIF)

Figure S4 **GltD expression is down-regulated during sporulation.** (A) Detection of GltD by Western blotting during a sporulation time course using GltD-specific antibodies. Extracts from a *gltD* mutant are shown as a specificity control. A nonspecific cross-reactive specie is shown as a control for comparable protein loading in all lanes. (B) Quantifications of the relative amounts of detectable GltD protein of the experiment shown in (A).
(TIF)

Figure S5 **Tracking NfsD-mCherry at the surface of sporulating cells.** (A) Subpixel-resolution NfsD-mCherry foci tracking and methodology. Tracking of a rotating NfsD-mCherry cluster in the focal plane is shown. In this example, the NfsD-mCherry cluster is moving in the macroscope focal plane, and therefore the orthodromic distance can be directly inferred from the images (orange and green). When clusters move out of the focal plane, orthodromic distances are calculated from euclidian distances (purple). (B) Geometric projections used to calculate orthodromic distances from Euclidian distances.
(TIF)

Figure S6 **The MreB cytoskeleton is not required for the function of Agl-Nfs.** (A) Addition of A22 immediately after Glycerol induction blocks cell rounding of WT but not mreB_V323A_ cells. (B) Addition of A22 after cell rounding initiation does not block sporulation. (C) The rotation of NfsD-mCherry is not affected by the addition of A22. Scale bar = 1 µm.
(TIF)

Figure S7 **NfsD-mCherry movement depends on Agl motor activity in absence of Exo secretion.** (A) NfsD-mCherry movement in an *exoA aglQ* mutant. Time-lapse recording was obtained on 4-h-old sporulating cells. Scale bar = 1 µm. (B and C) NfsD-mCherry movement is abolished by CCCP in the *exoA* mutant.
(TIF)

Figure S8 **Individual** ***z*** **-sections of BstkA-sfGFP and NfsD-mCh localization in the cell shown in** **Figure 6E** **.**
(TIF)

Figure S9 **Modular architecture of Agl-Glt/Nfs machineries in bacteria.**
(TIF)

Movie S1 **Sporulation of** ***Myxococcus*** **cells in the microfluidic chamber.** Sporulation kinetics after addition of glycerol. The movie is accelerated by a factor of 720.
(AVI)

Movie S2 **Rotation of NfsD-mCherry.** The time-lapse recording was obtained on a 4-h-old sporulating cell. The movie is accelerated by a factor of 480.
(AVI)

Movie S3 **Rotation of NfsD-mCherry is abolished in presence of CCCP (1 mM).** The time-lapse recording was obtained on a 4-h-old sporulating cell. The movie is accelerated by a factor of 480.
(AVI)

Movie S4 **Co-rotation of NfsD-mCherry and GSLI-FITC on a sporulating cell.** The time-lapse recording was obtained on a 4-h-old sporulating cell. The movie is accelerated by a factor of 360.
(AVI)

Movie S5 **3D reconstruction of a spore expressed BtkA-sfGFP and stained by FM4-64.** The Z-stack recording was obtained on a 4-h-old sporulating cell. Each image corresponds to a 10° rotation of the cell.
(AVI)

Table S1 **Bioinformatic analysis of the Glt and the Nfs clusters.**
(DOCX)

Table S2 **Strains used in this study.**
(DOCX)

Table S3 **Primers used in this study.**
(DOCX)

Table S4 **Plasmids used in this study.**
(DOCX)

## Abbreviations

agl: adventurous gliding
CCCP: carbonyl cyanide-m-chlorophenylhydrazone
GFP: green fluorescent protein
glt: gliding transducer
GSL-I: griffonia (bandeiraea) simplicifolia lectin I
MreB: murein cluster B
nfs: necessary for sporulation
PG: peptidoglycan
pmf: proton motive force
sfGFP: super-folder green fluorescent protein
TEM: transmission electron microscopy
WT: wild type
