# Supplementary figures and images for: A Versatile Class of Cell Surface Directional Motors Gives Rise to Gliding Motility and Sporulation in Myxococcus xanthus
Source: PLoS Biol. 2013 Dec 10;11(12):e1001728. doi: 10.1371/journal.pbio.1001728 (PMC3858216; doi:10.1371/journal.pbio.1001728)

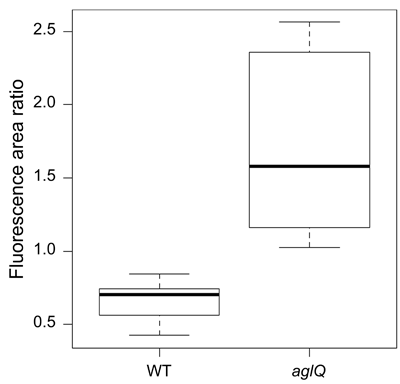

Supplement: Figure S1 — Loose anchoring of the main spore coat polymer in the aglQ mutant. Box plot representations of the ratio between the area of GSL-I fluorescence and a cell total area (fluorescence area ratio) are shown. For each strain, measurements were performed over 20 cells. (TIF) [file pbio.1001728.s001.tif]

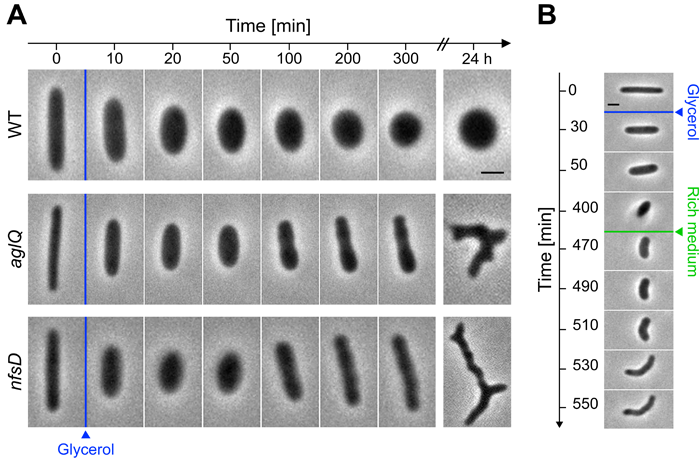

Supplement: Figure S2 — Sporulation of Myxococcus cells in the microfluidic chamber. (A) Sporulation kinetics after addition of glycerol. Following induction, cell rounding is observed with kinetics similar to cell rounding in liquid flasks. Aberrant cell shapes are observed both with the aglQ and nfsD mutants as described by [20]. Scale bar = 1 µm. (B) Germination after CYE (rich) medium injection. Spores germinate indicating that the observed round cells are indeed spores and not spheroplasts. (TIF) [file pbio.1001728.s002.tif]

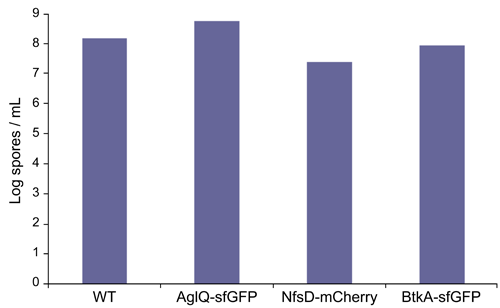

Supplement: Figure S3 — AglQ-sfGFP, NfsD-mCherry, and BtkA-sfGFP are fully functional for sporulation. Spore titers were determined and expressed as in Figure 2A. (TIF) [file pbio.1001728.s003.tif]

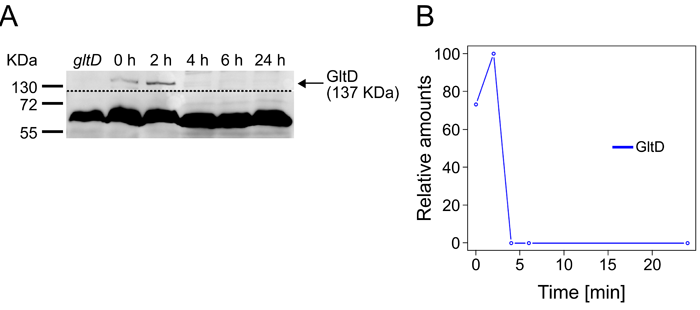

Supplement: Figure S4 — GltD expression is down-regulated during sporulation. (A) Detection of GltD by Western blotting during a sporulation time course using GltD-specific antibodies. Extracts from a gltD mutant are shown as a specificity control. A nonspecific cross-reactive specie is shown as a control for comparable protein loading in all lanes. (B) Quantifications of the relative amounts of detectable GltD protein of the experiment shown in (A). (TIF) [file pbio.1001728.s004.tif]

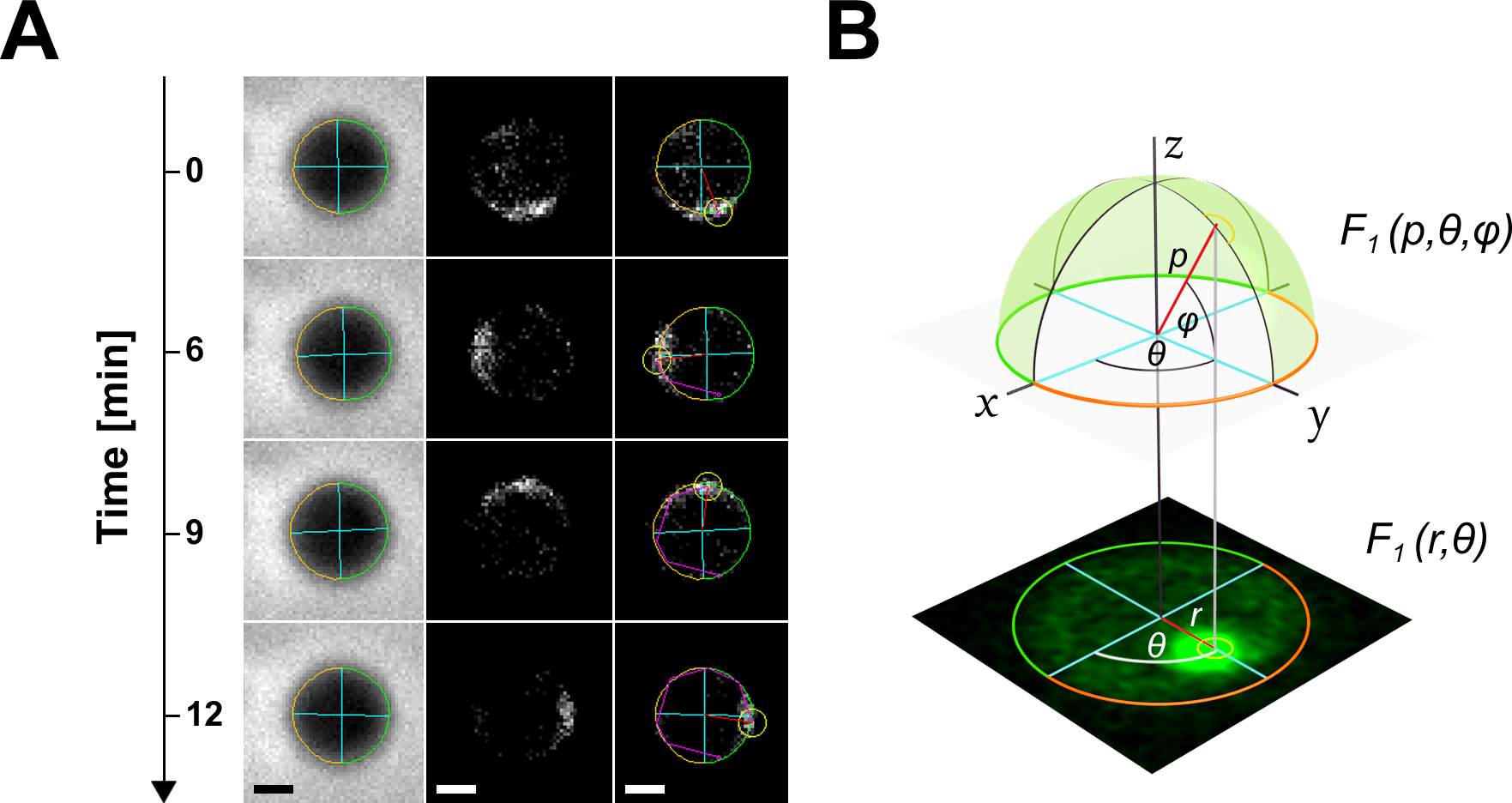

Supplement: Figure S5 — Tracking NfsD-mCherry at the surface of sporulating cells. (A) Subpixel-resolution NfsD-mCherry foci tracking and methodology. Tracking of a rotating NfsD-mCherry cluster in the focal plane is shown. In this example, the NfsD-mCherry cluster is moving in the macroscope focal plane, and therefore the orthodromic distance can be directly inferred from the images (orange and green). When clusters move out of the focal plane, orthodromic distances are calculated from euclidian distances (purple). (B) Geometric projections used to calculate orthodromic distances from Euclidian distances. (TIF) [file pbio.1001728.s005.tif]

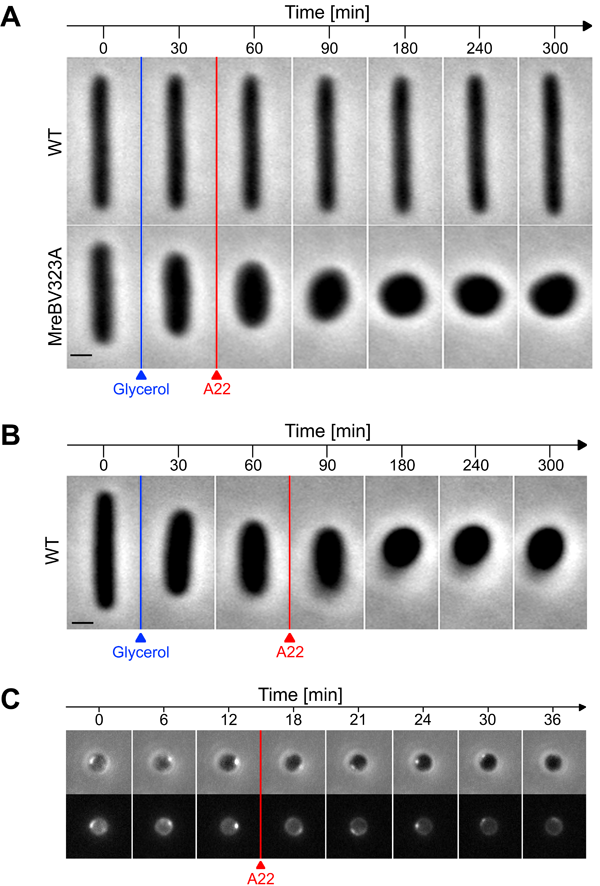

Supplement: Figure S6 — The MreB cytoskeleton is not required for the function of Agl-Nfs. (A) Addition of A22 immediately after Glycerol induction blocks cell rounding of WT but not mreBV323A cells. (B) Addition of A22 after cell rounding initiation does not block sporulation. (C) The rotation of NfsD-mCherry is not affected by the addition of A22. Scale bar = 1 µm. (TIF) [file pbio.1001728.s006.tif]

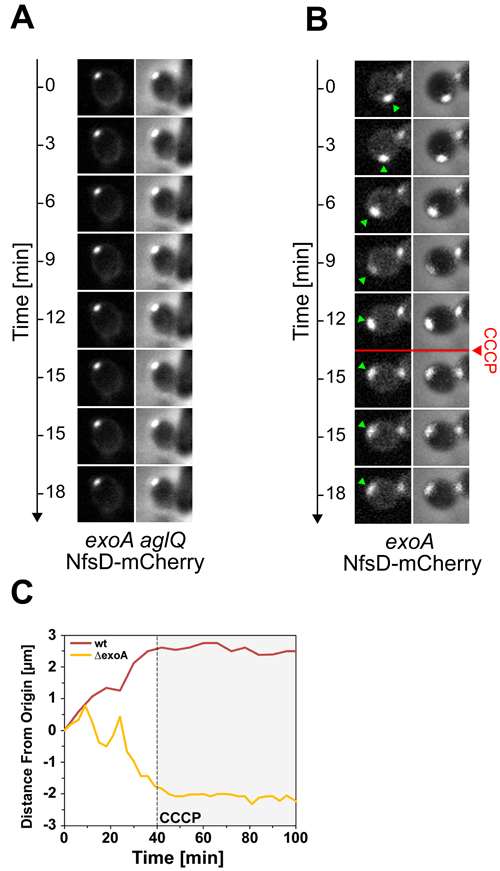

Supplement: Figure S7 — NfsD-mCherry movement depends on Agl motor activity in absence of Exo secretion. (A) NfsD-mCherry movement in an exoA aglQ mutant. Time-lapse recording was obtained on 4-h-old sporulating cells. Scale bar = 1 µm. (B and C) NfsD-mCherry movement is abolished by CCCP in the exoA mutant. (TIF) [file pbio.1001728.s007.tif]

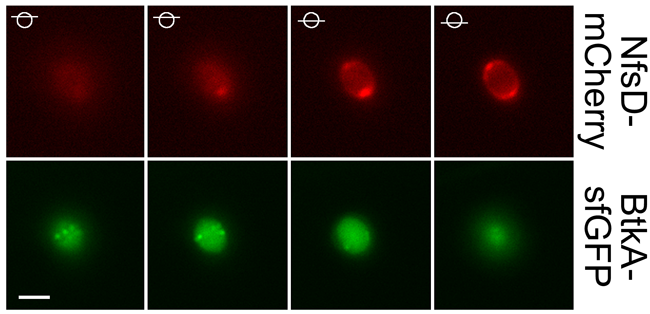

Supplement: Figure S8 — Individual z -sections of BstkA-sfGFP and NfsD-mCh localization in the cell shown in Figure 6E . (TIF) [file pbio.1001728.s008.tif]

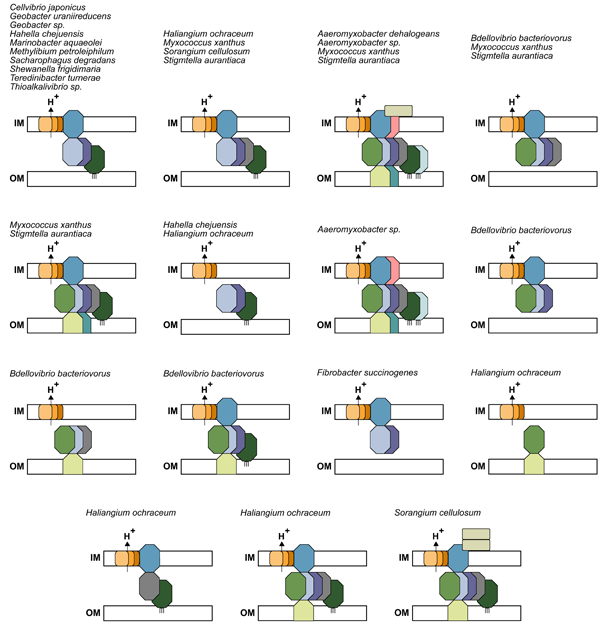

Supplement: Figure S9 — Modular architecture of Agl-Glt/Nfs machineries in bacteria. (TIF) [file pbio.1001728.s009.tif]
