# Supplementary material for: A Versatile Class of Cell Surface Directional Motors Gives Rise to Gliding Motility and Sporulation in Myxococcus xanthus
Source: PLoS Biol. 2013 Dec 10;11(12):e1001728. doi: 10.1371/journal.pbio.1001728 (PMC3858216; doi:10.1371/journal.pbio.1001728)
Supplement: Table S1 — Bioinformatic analysis of the Glt and the Nfs clusters. (DOCX) [file pbio.1001728.s015.docx]

**Table S1. Bioinformatic analysis of the Glt and the Nfs clusters.**

| **Protein in the Glt complex** | **Paralog in the Nfs complex** | **Identities** | **Positives** | **Functional domain (Pfam)** | **Signal peptide** | | **Transmembrane domain** | **Lipoprotein signal peptide** |
| --- | --- | --- | --- | --- | --- | --- | --- | --- |
| GltA (MXAN_2540) | NfsA (MXAN_3371) | 29 % | 43 % | OmpA-like transmembrane domain | + | - | | **-** |
| GltB (MXAN_2539) | NfsB (MXAN_3372) | 30 % | 46 % | - | + | - | | - |
| GltC (MXAN_2541) | NfsC (MXAN_3373) | 23 % | 45 % | Tetratricopeptide repeat | + | - | | - |
| GltD (MXAN_4870) | NfsD (MXAN_3374) | 28 % | 46 % | Tetratricopeptide repeat | + | - | | - |
| GltE (MXAN_4869) | NfsE (MXAN_3375) | 28 % | 47 % | Tetratricopeptide repeat | + | - | | + |
| GltF (MXAN_4868) | NfsF (MXAN_3376) | 42 % | 70 % | - | + | - | | - |
| GltG (MXAN_4867) | NfsG (MXAN_3377) | 35 % | 50 % | FHA domain Gram-negative; bacterial TonB protein | - | + | | - |
| GltH (MXAN_4866) | NfsH (MXAN_3378) | 28 % | 43 % | Autotransporter β-domain | + | - | | - |
| GltI (MXAN_4863) | - | - | - | Tetratricopeptide repeat | - | - | | - |
| GltJ (MXAN_4862) | - | - | - | - | - | + | | - |
| GltK (MXAN_2538) | - | - | - | - | + | - | | + |
