# Supplementary material for: A Versatile Class of Cell Surface Directional Motors Gives Rise to Gliding Motility and Sporulation in Myxococcus xanthus
Source: PLoS Biol. 2013 Dec 10;11(12):e1001728. doi: 10.1371/journal.pbio.1001728 (PMC3858216; doi:10.1371/journal.pbio.1001728)
Supplement: Table S2 — Strains used in this study. (DOCX) [file pbio.1001728.s016.docx]

**Table S2. Strains used in this study.**

| **Strain** | **Description** | **Source** | **Genotype** |
| --- | --- | --- | --- |
| DZ2 | Wild type | Laboratory collection | WT |
| TM146 | DZ2 Δ*aglQ* (pBJΔ*aglQ*) | [1] | Δ*aglQ* |
| TM247 | DZ2 *gltD-mCherry* (pBJ*gltD-mCherry*) | [2] | *gltD-mCherry* |
| TM312 | TM146 *mx8_att_::aglQ_D28N_-HA* (pSW*aglQ_D28N_-HA*) | [1] | Δ*aglQ aglQ_D28N_-HA* |
| TM357 | TM146 Δ*pilA* (pBJΔ*pilA*) | This work | Δ*aglQ* Δ*pilA* |
| TM363 | DZ2 Δ*3004* (pBJΔ*3004*) | [1] | Δ*3004* |
| TM384 | DZ2 Δ*aglR* (pBJΔ*aglR*) | [1] | Δ*aglR* |
| TM452 | DZ2 Δ*aglS* (pBJΔ*aglS*) | [1] | Δ*aglS* |
| TM484 | DZ2 Δ*exoA* (pBJΔ*exoA*) | [3] | Δ*exoA* |
| TM526 | DZ2 *nfsD-mCherry* (pBJ*nfsD-mCherry*) | This work | *nfsD-mCherry* |
| TM541 | TM357 *mx8_att_::aglQ-sfGFP* (pSW*aglQ-sfGFP*) | This work | Δ*aglQ* Δ*pilA aglQ-sfGFP* |
| TM578 | DZ2 Δ*nfsD* (pBJΔ*nfsD*) | This work | Δ*nfsD* |
| TM628 | TM541 *nfsD-mCherry* (pBJ*nfsD-mCherry*) | This work | Δ*aglQ* Δ*pilA aglQ-sfGFP nfsD-mCherry* |
| EC153 | Top 10 *T18-aglR* (pU*T18N-aglR*) | This work | *aglR-T18* |
| EC156 | Top 10 *T25-gltG* (pK*T25-gltG*) | This work | *T25-gltG* |
| EC176 | Top 10 *T25-nfsG* (pK*T25-nfsG*) | This work | *T25-nfsG* |
| EC208 | Top 10 *T18-3003* (pU*T18N-3003*) | This work | *3003-T18* |

1. Sun M, Wartel M, Cascales E, Shaevitz JW, Mignot T (2011) Motor-driven intracellular transport powers bacterial gliding motility. Proc Natl Acad Sci USA 108: 7559–7564.

2. Nan B, Mauriello EM, Sun IH, Wong A, Zusman DR (2010) A multi-protein complex from Myxococcus xanthus required for bacterial gliding motility. Mol Microbiol 76: 1539–1554.

3. Ducret A, Valignat M-P, Mouhamar F, Mignot T, Theodoly O (2012) Wet-surface-enhanced ellipsometric contrast microscopy identifies slime as a major adhesion factor during bacterial surface motility. Proc Natl Acad Sci USA 109: 10036–10041.
