# Supplementary material for: A Versatile Class of Cell Surface Directional Motors Gives Rise to Gliding Motility and Sporulation in Myxococcus xanthus
Source: PLoS Biol. 2013 Dec 10;11(12):e1001728. doi: 10.1371/journal.pbio.1001728 (PMC3858216; doi:10.1371/journal.pbio.1001728)
Supplement: Table S3 — Primers used in this study. (DOCX) [file pbio.1001728.s017.docx]

**Table S3. Primers used in this study.**

| **Primer** | **Sequences of primers (5’-3’)** |
| --- | --- |
| 3374-mCherry-1 | CCCAAGCTTGAGAAGGAGAAGAAGTGGAG |
| 3374-mCherry-2 | CTCGCCCTTGCTCACTCGCAGCACCTCCCCGCG |
| 3374-mCherry-3 | GGGGAGGTGCTGCGAGTGAGCAAGGGCGAGGAG |
| 3374-mCherry-4 | CGGGATCCTTACTTGTACAGCTCGTCC |
| 3374-mCherry-5 | CGGGATCCAGCTGTTTCGCATCGATTC |
| 3374-mCherry-6 | GGAATTCTCCAGCTTCAGGTAGACCATG |
| AglR-3 | CGGGATCCGAAGTCCTTCGGGAACCCG |
| GmoBSFGFP-1 | TTCTTCACCTTTAGAGCCCATCGCCGCGGACAC |
| GmoBSFGFP-2 | TCCGCGGCGATGGGCTCTAAAGGTGAAGAACTGTTC |
| GmoBSFGFP-3 | CCCAAGCTTTTATTTGTAGAGCTCATCCATG |
| D3374-1 | GGAATTCCGCTTATGAGCGAGTGCCG |
| D3374-2 | CGGGATCCGCGAGCTCCTGCCGTGAAG |
| D3374-3 | CGGGATCCAGCTGTTTCGCATCGATTC |
| D3374-4 | CCCAAGCTTACCTTCTCGAACGCCTCGC |
| gmoA-O1 | CCCAAGCTTGGACCTGGCGTCTGTGAC |
| gmoA-O2 | GGAATTCTCCTCCTCGTCGCGAG |
| 4867-O1 | GCTCTAGACGCCGTTCCTCTGACACTC |
| 4867-O2 | GGAATTCCTATTCGCCGGACTGCTTG |
| 3377-O1 | GCTCTAGACGCGGCGGCGAAGAACAAC |
| 3377-O2 | GGAATTCTCACCCTCCCGCGCCAGCG |
| 3003-O1 | CCCAAGCTTGATGGGCGCGCCCCG |
| 3003-O2 | GGAATTCCGCGAAGCGCGCGCCTC |
