# Supplementary material for: A Versatile Class of Cell Surface Directional Motors Gives Rise to Gliding Motility and Sporulation in Myxococcus xanthus
Source: PLoS Biol. 2013 Dec 10;11(12):e1001728. doi: 10.1371/journal.pbio.1001728 (PMC3858216; doi:10.1371/journal.pbio.1001728)
Supplement: Table S4 — Plasmids used in this study. (DOCX) [file pbio.1001728.s018.docx]

**Table S4. Plasmids used in this study.**

| **Plasmid** | **Construction scheme*** |
| --- | --- |
| pBJ114 *nfsD-mCherry* | Primer pairs 3374-mCherry-1/3374-mCherry-2 and 3374-mCherry-5/3374-mCherry-6 were used to amplify respectively the last 1 kb of *nfsD* gene (fragment 1) and the 1 kb downstream *nfsD* gene (fragment 3) from the DZ2 chromosome. Primer pair 3374-mCherry-3/3374-mCherry-4 is used to amplify *mCherry* from a plasmid containing the *mCherry* gene (fragment 2). Fragments 1 and 2 were fused by SOE-PCR and cloned at the HindIII and BamHI restriction sites of the pBJ114 (pBJ114-fragment1-2). Then, the fragment 3 is cloned at the BamHI and EcoRI restriction sites of the pBJ114-fragment1-2. |
| pSWU30 *aglQ-sfGFP* | Primer pair AglR-3/GmoBSFGFP-1 is used to amplify the *aglQ* gene and its promoter from the DZ2 chromosome (fragment 1). Primer pair GmoBSFGFP-2/ GmoBSFGFP-3 is used to amplify the *sfGFP* from a plasmid containing the *sfGFP* gene (fragment 2). Both fragments fused by SOE-PCR and cloned at the BamHI and HindIII restriction sites of the pSWU30. |
| pBJΔ*nfsD* | Primer pairs D3374-1/ D3374-2 and D3374-3/ D3374-4 were used to amplify respectively a 1 kb fragment upstream and downstream of the *nfsD* open reading frame. The upstream fragment was first cloned at the EcoRI and BamHI restriction sites of the pBJ114 (pBJ114-upstream). Then, the downstream fragment was cloned at the BamHI and HindIII restriction sites of the pBJ114-upstream. |
| pU*T18N-aglR* | A fragment encompassing *aglR* was amplified from the DZ2 chromosome with primers gmoA-O1/gmoA-O2 and cloned at the HindIII and EcoRI sites of pUT18N. |
| pK*T25-gltG* | A fragment encompassing *gltG* was amplified from the DZ2 chromosome with primers 4867-O1/4867-O2 and cloned at the XbaI and EcoRI sites of pKT25. |
| pK*T25-nfsG* | A fragment encompassing *nfsG* was amplified from the DZ2 chromosome with primers 3377-O1/3377-O2 and cloned at the XbaI and EcoRI sites of pKT25. |
| pU*T18N-3003* | A fragment encompassing *3003* was amplified from the DZ2 chromosome with primers 3003-O1/3003-O2 and cloned at the HindIII and EcoRI sites of pUT18N. |

*All plasmid inserts were sequenced to ensure the absence of PCR-introduced mutations.
